# Tinetti balance performance is associated with mortality in older adults with late-onset Parkinson’s disease: a longitudinal study

**DOI:** 10.1186/s12877-023-03776-7

**Published:** 2023-01-30

**Authors:** Louise Laurent, Pierre Koskas, Janina Estrada, Mélanie Sebbagh, Sophie Lacaille, Agathe Raynaud-Simon, Matthieu Lilamand

**Affiliations:** 1 Assistance Publique-Hôpitaux de Paris.Nord, Bretonneau University Hospital, Geriatric day hospital, 23 rue Joseph de Maistre, 75018 Paris, France; 2 Université Paris Cité, Paris, France; 3 INSERM UMR-S 1144 research unit, Paris, France; 4 Assistance Publique-Hôpitaux de Paris.Nord, Lariboisière-Fernand Widal, Geriatric department, 200 rue du Fbg St Denis, 75010 Paris, France

**Keywords:** Parkinson’s disease, Older adults, Tinetti, Balance

## Abstract

**Background:**

Parkinson’s disease (PD) is associated with a 3-fold mortality risk, which is closely related to advancing age. Evidence is lacking regarding the factors associated with the risks of mortality or nursing-home (NH) admission, in elderly patients with PD. We aimed at identifying the clinical characteristics associated with these outcomes, in older community-dwelling patients with late-onset PD.

**Methods:**

Retrospective, observational analysis of data from geriatric day hospital patients. Motor assessment included Unified Parkinson Disease Rating Scale (UPDRS) part III score, Tinetti Performance Oriented Mobility Assessment (POMA) balance and gait tests, and gait speed. Levodopa equivalent dose, comorbidity, cognitive performance, Activities of Daily Living performance were examined. Cox proportional hazards models were performed to identify the factors associated with mortality and NH admission rate (maximum follow-up time = 5 years).

**Results:**

We included 98 patients, mean age 79.4 (SD = 5.3) of whom 18 (18.3%) died and 19 (19.4%) were admitted into NH, over a median follow-up of 4 years. In multivariate Cox models, poor balance on the Tinetti POMA scale (HR = 0.82 95%CI (0.66–0.96), *p* = .023) and older age (HR = 1.12 95%CI (1.01–1.25), *p* = .044) were the only variables significantly associated with increased mortality risk. A Tinetti balance score below 11/16 was associated with a 6.7 hazard for mortality (*p* = .006). No specific factor was associated with NH admissions.

**Conclusions:**

Age and the Tinetti POMA score were the only factors independently associated with mortality. The Tinetti POMA scale should be considered for balance assessment and as a screening tool for the most at-risk individuals, in this population.

**Supplementary Information:**

The online version contains supplementary material available at 10.1186/s12877-023-03776-7.

## Introduction

Parkinson’s disease (PD) is the second most common neurodegenerative disease after Alzheimer’s Disease, and represents the leading cause of motor disability in older adults [1]. More than half of the patients with PD are older than 75, and this disease affects up to 3.5% of the population aged over than 80 [1, 2]. Multimorbidity and non-motor symptoms, which are common in the elderly, worsen motor function decline and increase the mortality in PD [3, 4].

Subjects with PD are likely to experience a shorter life span compared to healthy individuals of the same age, and this risk of dying increases with aging [5–7]. Thus, 90% of deaths in PD patients occur after the age of 70, after at least 10 years of disease progression [8]. The presence of major neurocognitive disorders and the incidence of falls increase this risk [1, 5]. Impaired swallowing and dysphagia, leading to pneumonia, also shorten the lifespan of individuals with PD [9]. The American Academy of Neurology has highlighted higher age at onset of PD, associated comorbidities, presentation with rigidity and bradykinesia, and secondary decrease in dopaminergic sensitivity, as predictors of faster motor progression and increased mortality [5, 10]. Other studies also showed that mortality in PD was independently associated with motor disability, low motor Unified Parkinson Disease Rating Scale (UPDRS) score, as well as the presence of psychotic symptoms [6, 8].

Individuals with PD might also face a greater risk of limitations in daily living activities and nursing-home (NH) admission, compared to the general population, although evidence remains heterogeneous and controversial [11, 12]. Women with PD were shown to be five times more likely admitted to a NH than men [13]. Progression of motor symptoms, cognitive impairment and neuropsychiatric disorders (delusion and hallucinations), disability and older age have been associated with increased odds of NH admission [12, 14–16]. Non-motor symptoms, iatrogenic conditions and polypharmacy have also been related to poor prognosis in elderly patients with PD [1, 4, 17].

However, there is limited available evidence relating to the predictors of survival and nursing home (NH) admission, in adults older than 70 years with late-onset Parkinson’s disease (PD), as they are less likely to participate in clinical research [18]. More in-depth knowledge of these factors is crucial to help develop personalized care plans for the most at-risk individuals, or to design future clinical trials to improve these outcomes. The aim of this study was to identify the clinical characteristics of community dwelling older patients with late-onset PD, associated with survival status and NH admission.

## Methods

### Design and setting

This is a retrospective, monocentric, observational analysis of data from community- dwelling older adults with PD, admitted between July 2007 and March 2019, in the geriatric day hospital of Bretonneau (Assistance Publique-Hôpitaux de Paris. Nord, France). This hospital is a referral center for geriatric care in the northern Parisian district. PD diagnosis was based on clinical examination by the referring neurologist, positive response to Levodopa treatment [10] and clinical confirmation by the hospital neurologist.

### Eligibility criteria

#### Inclusion criteria were

- Age at PD diagnosis of 65 years and older (i.e. late-onset PD),
- Comprehensive assessment performed by our multidisciplinary day hospital staff (geriatrician, neurologist and physiotherapists)

#### Exclusion criteria were:

- Absence of available follow-up information for at least 12 months after the first evaluation
- Presence of clinical symptoms suggestive of others Parkinson syndromes, according to the American Academy of Neurology recommendations [10]
- Evidence for another neurodegenerative disease (e.g. Alzheimer’s disease), at the time of the first evaluation or during the period of follow-up
- Absence of consent to the use of their medical data
- Insufficient fluency in French to understand and perform the tests or legal guardianship

### Clinical assessment

All patients underwent an initial comprehensive geriatric assessment along their first stay in the day hospital including as follows:

- Age at first day hospital assessment, gender,
- PD duration,
- Number and severity of comorbidities, using the Cumulative Illness Rating Scale (CIRS-G scale, score 0–56) [19],
- Performance in Activities of Daily Living (ADL) (score 0–6) [20] and instrumental activities of daily living (IADL) (four items from the Lawton’s scale: *ability to use telephone*; *mode of transportation*; *responsibility for own medications*; *ability to handle finances*; score 0–4) [21],
- Cognitive performance according to the Mini Mental State Examination (MMSE) (score 0–30) [22],
- Weight measurement and Body Mass Index calculation,
- Handgrip strength of the dominant hand, measured by hydraulic dynamometer (Sissel, Sweden)
- Number of drugs taken per day and daily Levodopa equivalent dose calculated (for conversing factors used, see Schade et al. [23]).

### Motor assessment

- A physiotherapist performed the following tests for all included participants:
  - UPDRS part III score (0–108) [24] and UPDRS axial sub-score, defined as the sum of the following motor subscores: speech, rigidity of neck, arising from chair, gait, postural stability and posture (items 18, 22, 27, 28, 29 and 30) of the UPDRS Part III (score 0–24) [25].
  - Tinetti Performance Oriented Mobility Assessment (POMA) balance (score 0–16) and gait (score 0–12) tests; higher scores on Tinetti balance and gait subscales indicating better performance [26].
  - Timed Up and Go (TUG) and Cognitive Timed Up and Go (same test while performing a cognitive dual task: counting backwards from 100 by 2). Both tests were timed in seconds [27].
  - Gait speed was measured on a 10-m distance, asking the patient to walk at his/her usual pace, starting from a standing still position, with or without the use of auxiliary aids. Time was measured with a digital stopwatch, from when instructed to start until crossing the 10-m marking.

### Main outcomes

The primary endpoint of the study was survival status (all-cause mortality). The secondary endpoint was NH admission.

Survival status and housing status (i.e. occurrence of NH admission) were retrospectively collected every 6 months, over a maximum follow-up period of 5 years, using data available on the professional medical database of the hospital (ORBIS® software), completed by phone interviews with the patients, their general physician, specialists or staff of rehabilitation care units or NH. The date of data censoring was set as March 1st 2020.

### Statistical analysis

Demographic and clinical characteristics of patients at inclusion were subjected to descriptive statistics. Continuous variables were compared according to the survival status and to the housing status of patients during follow-up, and the differences between groups were calculated using Student’s t-test and Wilcoxon test, and by Fischer’s exact test for gender, the only categorical variable. The threshold of statistical significance was set for *p* values less than 0.05. Variables showing an association with mortality with a significance level of *p* < 0.2 in univariate analysis were analyzed using a multivariate Cox proportional hazard model and hazard ratios are reported with a 95% confidence interval. Data from patients lost to follow-up were censored. All statistical analyses were performed using R software (version 1.2.5).

### Ethical considerations and data sharing policy

The research database was approved by the National Commission for Information Technology and Civil Liberties (Commission Nationale de l’Informatique et des Libertés, record number 1858079), since 2006. Consistent with the European General Data Protection Regulation, all patients were provided written and oral information about the data management explaining how their health data could be used anonymously for clinical research. The study protocol was also approved by the Regional Ethics Committee in Geriatric Medicine (CEGID Gérond’if, Paris 2021 #12021). According to the latter statements, despite full data availability in our records, sharing the clinical data of our patients would compromise the ethical standards mentioned above.

## Results

### Study population

**Fig. 1:**
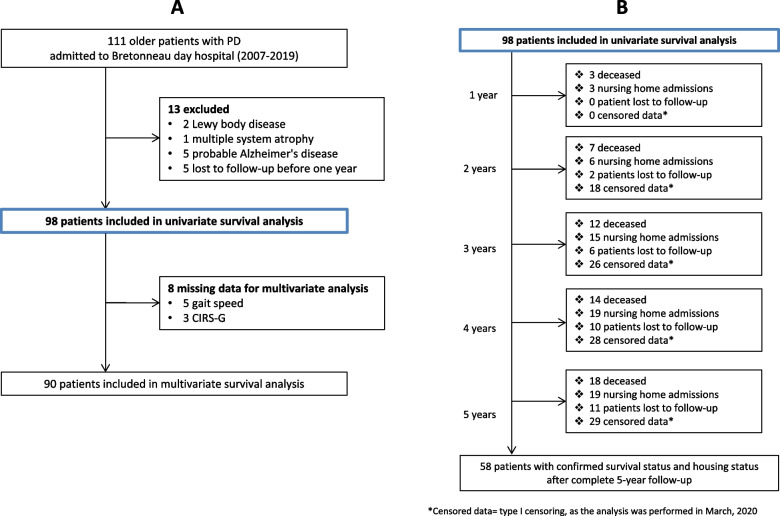
Flow chart of study participants (**A**) and cumulative number of events by year, during 5-year monitoring (**B**)

One hundred eleven patients with an initial diagnosis of PD were referred to Bretonneau day hospital between July 2007 and March 2019. Thirteen patients were excluded from our analysis (See flow-chart on Fig. 1A). Therefore, we included 98 participants in our univariate analyses.

The mean follow-up duration was 3.4 (standard deviation = 1.7) years. Survival status and housing status (i.e. occurrence or not of NH admission) during follow-up was confirmed in 58 patients (59.2%), after complete 5-year monitoring. Point censoring occurred for 29 patients (29.6%) included after March 2015, when death or NH admission were not observed within the study duration (less than 4 years). Twenty-nine patients (29.6%) were lost to follow-up between 1 and 5 years after inclusion (Fig. 1B).

**Fig. 2:**
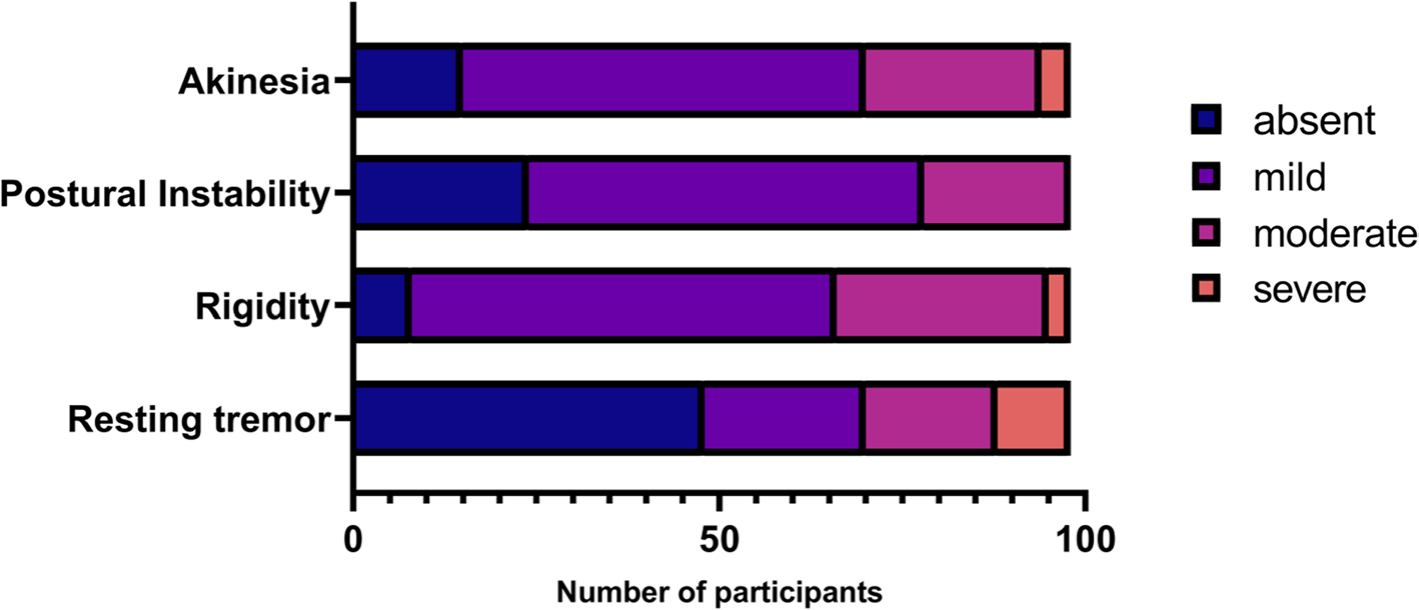
Main clinical symptoms of the study participants at baseline

Mean age was 79.4 (5.3), ranging from 68 to 94 years old. The mean PD duration was 3.8 (3.6) years. Our sample comprised 57% of males. Few patients had severe motor impairment; rigidity, bradykinesia and postural instability were common, whereas resting tremor was absent in half of the population (Fig. 1). Eighteen (18.4%) patients died during the follow up period, with a mean age at death of 84.5 years. The detailed characteristics of the study population are displayed in Table 1. The motor symptoms of the participants are described in Fig. 2.

**Table 1:** Patients’ characteristics, according to their survival status or nursing home admission over follow-up

Characteristics M (SD)	Full sample (***N*** = 98)	Survival status			Nursing home admission		
		Alive ***N*** = 80	Deceased ***N*** = 18	***P value***	No ***N*** = 79	Yes ***N*** = 19	***P value***
Age (years)	79.4 (5.3)	78.9 (5.2)	81.6 (5.4)	.07	79.4 (5.4)	79.6 (5.2)	.84
Male gender % (N)	57.1 (56)	52.5 (42)	77.8 (14)	.07	57.0 (45)	57.9 (11)	1.00
PD duration (Y)	3.8 (3.6)	3.8 (3.7)	3.7 (3.1)	.85	3.7 (3.7)	4.2 (3.1)	.59
UPDRS part III score [0–108]	14.6 (5.1)	14.5 (4.8)	15.2 (6.4)	.68	14.8 (5.1)	14.1 (5.1)	.62
UPDRS axial score [0–24]	7.9 (3.3)	7.8 (3.1)	8.3 (4.0)	.54	7.9 (3.3)	7.6 (3.4)	.68
Levodopa equivalent dose (mg)	368.5 (212.0)	368.5 (224.3)	368.8 (143.6)	1.00	364.9 (210.1)	385.9 (226.9)	.72
Weight (kg)	65.1 (11.7)	64.8 (11.8)	66.4 (11.5)	.66	65.4 (12.1)	63.9 (10.5)	.62
MMSE [0–30]	25.6 (3.8)	25.8 (3.5)	24.8 (5.4)	.53	25.7 (3.8)	25.1 (4.1)	.65
Grip strength (kg)	22.7 (8.0)	23.1 (8.2)	20.6 (6.5)	.25	22.9 (8.4)	21.8 (5.6)	.59
ADL score [0–6]	5.5 (0.8)	5.5 (0.9)	5.6 (0.7)	.80	5.5 (0.8)	5.6 (0.8)	.70
IADL score [0–4]	3.3 (1.1)	3.3 (1.0)	3.1 (1.2)	.38	3.2 (1.1)	3.2 (1.0)	.75
**Gait speed (m/s)**	**0.8 (0.3)**	**0.8 (0.3)**	**0.7 (0.3)**	**.038**	0.8 (0.2)	0.8 (0.4)	.81
TUG (s)	18.1 (9.2)	17.5 (9.0)	20.6 (9.8)	.24	18.1 (9.5)	18.0 (8.2)	.94
TUGc (s)	24.9 (15.7)	24.4 (15.2)	27.0 (18.2)	.57	24.5 (15.7)	26.1 (16.0)	.51
**Tinetti balance score [0–16]**	**13.3 (2.7)**	**13.7 (2.1)**	**11.6 (4.0)**	**.002**	13.4 (2.6)	13.2 (2.5)	.70
Tinetti gait score [0–12]	9.1 (2.7)	9.2 (2.7)	8.6 (2.6)	.42	9.1 (2.8)	9.2 (2.1)	.90
Number of drugs	5.1 (2.7)	5.1 (2.7)	5.3 (2.7)	.72	5.1 (2.8)	5.4 (2.3)	.55
CIRS-G score [0–56]	6.4 (2.1)	6.2 (2.0)	7.2 (2.5)	.13	6.4 (2.2)	6.4 (1.8)	1.00

*Abbreviations*: *ADL* Activities of Daily Living CIRS-G = Cumulative Illness Rating Scale for Geriatrics, *IADL* Instrumental Activities of Daily Living, *M* Mean, *MMSE* Mini Mental State Evaluation, *N* Number of subjects, *SD* Standard deviation, *TUG* Timed Up and Go test, *TUGc* Timed Up and Go Cognitive task, *UPDRS* United Parkinson’s Disease Rating Scale, *Y* Years

### Factors associated with mortality

In univariate analyze (Table 1), a slower gait speed at baseline was significantly associated with higher mortality (*p* = .038). Tinetti balance score, on admission, was also lower in deceased participants than in those who survived (11.6 vs. 13.6, *p* = .002).

**Fig. 3:**
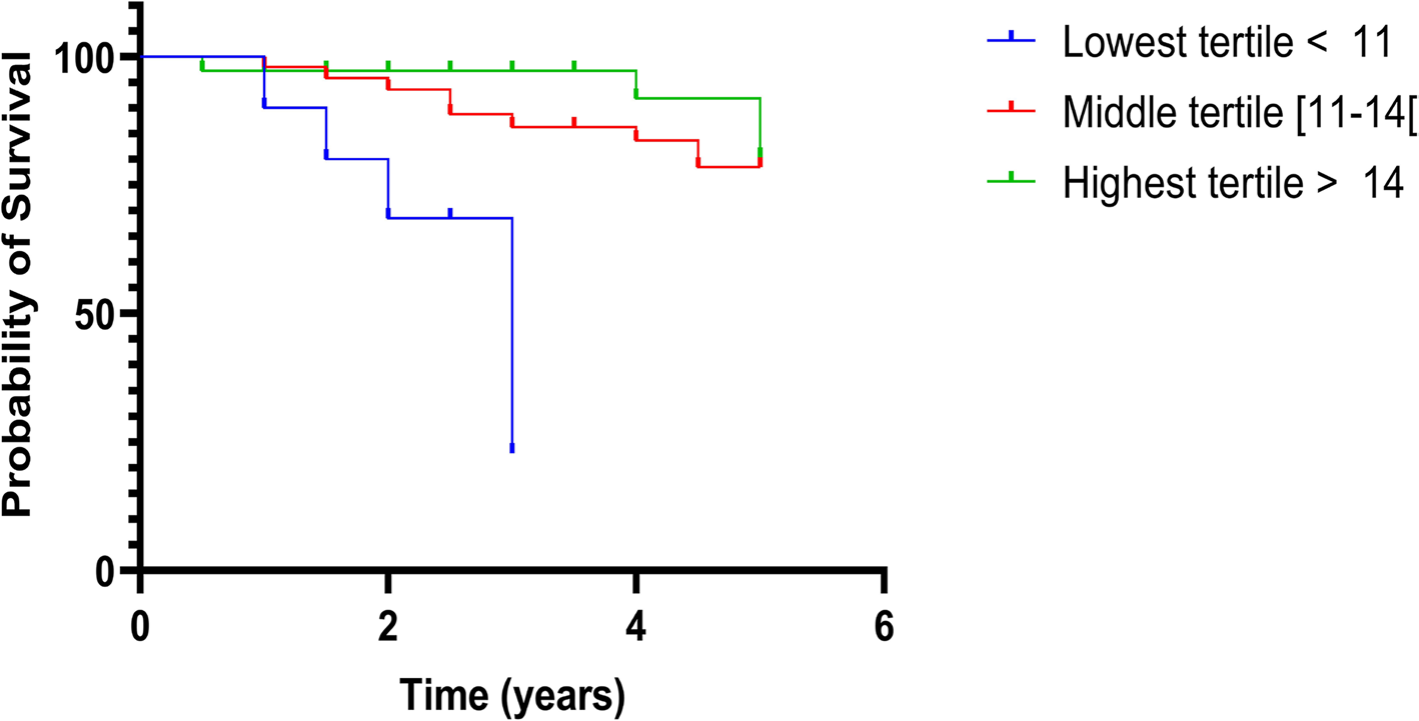
Survival probability according to the Tinetti Performance Oriented Mobility Assessment performance, in tertiles

After exclusion of eight subjects from our initial sample, due to missing data, 90 patients were included in the multivariate Cox model (Table 2). The only factor different between the two groups was participants’ abilities for basic ADL, which was not related to survival nor NH admission rate (See supplementary table). In our multivariate analysis, two factors remained independently associated with mortality: age and Tinetti balance score (HR = 0.82 95% CI [0.66–0.96] *p* = .023). To compare the mortality risk of patients with poor balance to those with normal balance skills, we performed a post-hoc multivariate Cox proportional hazard model analysis, including age and gender, considering the Tinetti balance score in tertiles (Table 3). Individuals from the lowest tertile (score below 11/16) experienced a six-fold higher mortality rate than those from the highest tertile (score over 14/16) (HR 6.65 95% CI [1.68–26.30], *p* = .006), See Table 3. Kaplan-Meier survival curves, according to the Tinetti balance score tertiles are presented in Fig. 3.

**Table 2:** Multivariate analysis of factors associated with mortality

Characteristics	HR (CI 95%)	***p*** value
Age (years)	1.12 (1.01–1.25)	**.044**
Male gender	3.06 (0.95–9.84)	.06
CIRS-G	1.04 (0.83–1.29)	.74
Gait speed (m/s)	0.44 (0.02–8.49)	.59
Tinetti balance score /16	0.82 (0.66–0.96)	**.023**

*N* = 90; 17 events

*CI* Confidence interval, *CIRS-G* Cumulative Illness Rate Scale for Geriatrics, *HR* Hazard Ratio

**Table 3:** Multivariate analysis of factors associated with mortality including tertiles of Tinetti POMA balance performance

Characteristics	HR (CI 95%)	*P*
Age (years)	1.07 (0.98–1.17)	.14
Male gender	2.78 (0.89–8.70)	.08
Tinetti balance score /16		
- First tertile (> 14)	REF	REF
- Second tertile ([11;14[)	1.26 (0.37–4.28)	.70
- Third tertile (< 11)	6.65 (1.68–26.3)	**.006**

*N* = 90; 17 events

*Abbreviations*: *CI* Confidence interval, *HR* Hazard Ratio

### Post-hoc analyses of the items of the Tinetti POMA balance scale

All the single items of the Tinetti balance test were included in a multivariate model to explore their association with mortality (data not shown). The *ability to maintain balance with eyes closed* was associated with a significant reduction in mortality HR = 0.26 [0.07–0.95, *p* = .038), with a sensitivity of 22.2% (CI 6.41–47.64%) and a specificity of 93.75% (CI 86.01–97.94%). This association was no longer significant after Bonferroni correction for multiple comparisons.

### Factors associated with nursing home admission

A total of 19 patients (19.4%) were admitted to a NH, within the study duration. No single factor was significantly associated with the odds of NH admission, in our study.

## Discussion

In this retrospective, observational study of older patients with late-onset PD, we observed a significant association between poor performance on the Tinetti POMA balance test and increased mortality. Moreover, individuals from the lowest tertile (score of less than 11/16) experienced 6-fold higher mortality than their counterparts with the best performance (score greater than 14/16). This association was independent of age, gender or multimorbidity. No other single factor was associated with mortality risk or NH admission rate, at the end of follow-up.

To our knowledge, there are no risk-assessment tools for clinicians to identify older patients with PD at risk of poor clinical outcomes, such as mortality or NH admission. Although the comprehensive assessment of motor and cognitive performances play a key role in routine assessment, none of them demonstrated a predictive value for adverse outcomes in this population [28, 29]. In our study, the 5-year mortality was 18.3% and the time lapse between diagnosis of PD and death ranged from 1.5 to 13.5 years, reflecting a major clinical heterogeneity, which was already described elsewhere [7, 30]. Nonetheless, this mean duration of 6.9 years in our analysis was shorter than in other published studies (on average, 12 years from the onset of the disease) [31, 32]. This difference may reflect the faster adverse consequences of PD symptoms, in elderly patients, compared with younger ones [31, 33].

The severity of motor impairment was suggested as a prognostic factor for disease progression, disability or impaired quality of life in several studies [10, 11, 34]. However, the most used tool in this setting is the UPDRS part III score, which was not associated with mortality in our study of elderly subjects with PD. Axial motor impairment alters mobility and was also proposed as a prognostic factor, especially in late-onset PD [33]. Yet, the measured axial UPDRS sub-score was not associated with mortality in our sample. Although the UPDRS part III score remains essential in the follow-up of patients with PD, our results highlighted the role of other tests in the clinical evaluation of patients with this disease. In our analysis, a poor Tinetti balance (but not gait) performance was significantly associated with higher mortality. Another study suggested a link between reduced physical function assessed with the Tinetti test and an increased mortality risk in individuals with PD [34]. However, the latter study included younger patients with PD (mean age 74.0 vs. 79.4 in our study) and did not dissociate the two subparts of the Tinetti Performance Oriented Mobility Assessment. Thus, our results underlined the importance of static balance disorder in elderly patients with PD [35]. Although gait, balance and posture disorders frequently coexist, they are not systematically associated, and when they are, one disorder may be dominant. A major issue in the care planning of individuals with PD is to identify modifiable risk factors to help prevent mortality or other adverse events (e.g. falls, disability), improve patients’ quality of life, and alleviate the overall public heath burden of the disease. In this context, the postural instability and gait dysfunction (PIGD) phenotype has been associated with faster PD progression as well as with a greater risk cognitive impairment, which in turn could increase the risks of functional decline, NH admission or mortality [36, 37]. An accurate evaluation of balance, with objective and quantitative measures in patients with PD, should be mandatory to support the prescription of a specific rehabilitative training. However, balance are often tested using the clinical pull test, which has a low inter-rater reliability and gives little insights into the patient’s balance in everyday life [38, 39]. However, the Tinetti balance test can be performed in less than 5 minutes, in daily clinical practice. A score below 11/16 is likely to indicate a substantially higher mortality risk and should encourage the neurologist or the geriatrician to propose a reinforced individualized treatment plan, to minimize the risk of falls, and which should include physical activity, physiotherapy and rehabilitation.

We did not find any other factor associated with mortality in our population. In our multivariate model, gait speed, although significantly lower in participants who died during the follow-up period in our study, was no longer significantly associated with mortality. The interaction between the Tinetti score and gait speed was non-significant (*p* = 0.19), despite a significant correlation between the two variables (*r* = 0.52 *p* < 0.001). However, several studies supported an association between gait speed and survival in elderly subjects [40, 41]. Our population included individuals with slow gait speed (mean = 0.8 m/s), most of whom could be considered at risk of falling. We assume that as gait speed was globally slow in our population of older patients with PD, as a consequence of the disease but also due to multimorbidity and advancing age. Therefore, this measure may have a limited predictive value for mortality in our population, although it was established in the general population [42].

Except older age, other non-physical characteristics: gender, cognitive performance, daily living ability, medication (including levodopa equivalent prescribed dose) were not associated with mortality risk in our study population. There is conflicting evidence in the literature regarding the influence of gender on disease progression [13, 43]. Cognitive status, as reflected by the MMSE score, was generally good in our patients, with a mean score above 25/30 at inclusion. Evidence from the literature suggested that the MMSE score was insignificantly altered in the early stages of PD [44]. The daily living abilities of the patients included in our study, as indicated by the ADL and IADL scores, was also globally preserved, probably explaining the absence of a significant association with mortality risk. Likewise, other studies did not find any association between the initial dose of levodopa and mortality risk [17].

We did not find any single factor associated with the risk of NH admission. This result is in contrast with results from Hosking et al. who identified, in NH residents with parkinsonism, a higher symptom burden, in particular cognitive impairment, increased disease severity on the UPDRS scale or disability compared to patients with a similar condition but living in their own home [16]. However, unlike in our study, the former one had a cross-sectional design and involved patients with late-stage parkinsonism. NH admission depends on multiple factors, beyond the mere consequences of motor or cognitive decline. Evidence regarding the causal factors of NH admission in PD is controversial [45, 46]. In particular, home care support, either family or professional, can enable disabled patients to remain at home. Social and financial situation factors, which were not evaluated in our study, also play a key role in patients’ outcomes. Conversely, social isolation or low income may result in a faster admission to a NH.

The main strength of our study is that, to our knowledge, it is the first one carried out specifically in elderly PD patients, focusing on factors associated with survival status or NH admission rate, and using data systematically recorded for more than 10 years. However, our study also has several limitations such as the fact that there was a limited number of events (deaths and NH admissions) due to the small size of our population, which potentially undermines its power. The inclusion criterion of an age at diagnosis of 65 and older may have skewed the results, since late-onset PD per se is an established mortality risk factor [47]. The underlying causes of death were not known and could have been associated with independent comorbidities. The motor assessment using the UPDRS part III scale, developed in the 1980’s might be refined by the revised Movement Disorder Society- UPDRS scale [48], which was not available when our first participants where included. Furthermore, this was a retrospective study, with more than 10% of participants lost to follow-up. Nearly 30% of our patients had a shorter follow-up period as they were included less than 5 years before the end of the collection period. However, our results indicate the increased odds of dying, in individuals with the poorest performance on the Tinetti balance scale.

## Conclusion

Older individuals with late-onset PD experience various patterns of motor and non-motor symptoms associated with an overall increased mortality risk. Among multiple assessment tools, the Tinetti POMA test may be an accurate instrument for identifying the older individuals with late-onset PD at higher risk of dying. This instrument should be considered in clinical practice, both by physiotherapists and geriatricians, to improve the care of older adults with late-onset PD. Future prospective studies are warranted to confirm the predictive value of the Tinetti POMA in this population.

## Supplementary Information

**Additional file 1: Supplementary table.** Comparison of the characteristics of patients included in the multivariate analysis vs those excluded.

## Data Availability

The datasets generated and analysed during the current study are not publicly available, to preserve the confidentiality of study participants and the data collected from them, consistent with the information note, but are available from the corresponding author, on reasonable request.
