# Supplementary material for: Tinetti balance performance is associated with mortality in older adults with late-onset Parkinson’s disease: a longitudinal study
Source: BMC Geriatr. 2023 Jan 30;23:54. doi: 10.1186/s12877-023-03776-7 (PMC9887890; doi:10.1186/s12877-023-03776-7)
Supplement: Supplementary file 1 — Additional file 1: Supplementary table. Comparison of the characteristics of patients included in the multivariate analysis vs those excluded. [file 12877_2023_3776_MOESM1_ESM.docx]

| **Characteristics**  **M (SD)** | **Study population**  **(univariate analysis)**  **(N=98)** | **Population of the multivariate Cox analysis**  **(N=90)** | **Individuals excluded from the Cox analysis**  **(N=8)** | **p** |
| --- | --- | --- | --- | --- |
| Age (years) | 79.4 (5.3) | 79.6 (5.2) | 77.3 (6.8) | 0.23 |
| Male gender % (N) | 57.1 (56) | 56.6 (51) | 62.5 (5) | 1.00^a^ |
| Time to onset (Y) | 3.8 (3.6) | 3.8 (2.3) | 3.8 (3.7) | 0.97 |
| UPDRS part III score [0-108] | 14.6 (5.1) | 14.6 (4.9) | 15.0 (7.1) | 0.83 |
| UPDRS axial score [0-24] | 7.9 (3.3) | 7.9 (3.1) | 7.8 (4.7) | 0.92 |
| Levodopa equivalent dose (mg) | 368.5 (212.0) | 362.0 (213.0) | 438.0 (205.0) | 0.34 |
| Weight (kg) | 65.1 (11.7) | 65.2 (12.0) | 64.0 (8.4) | 0.81 |
| MMSE [0-30] | 25.6 (3.8) | 25.5 (3.8) | 27.2 (4.0) | 0.13^b^ |
| Grip strength (kg) | 22.7 (8.0) | 22.6 (7.7) | 23.9 (11.2) | 0.69 |
| **ADL score [0-6]** | 5.5 (0.8) | 5.6 (0.7) | 4.8 (1.4) | **<0.01** |
| IADL score [0-4] | 3.3 (1.1) | 3.3 (1.0) | 2.8 (1.5) | 0.14 |
| Gait speed (m/s) | 0.8 (0.3) | 0.8 (0.3) | 0.9 (0.2)^c^ | 0.50^b^ |
| TUG (s) | 18.1 (9.2) | 17.9 (8.8) | 19.5 (13.5) | 0.65 |
| TUGc (s) | 24.9 (15.7) | 24.3 (12.6) | 30.9 (36.6) | 0.26 |
| Tinetti balance score [0-16] | 13.3 (2.7) | 13.4 (2.6) | 12.3 (3.2) | 0.23 |
| Tinetti gait score [0-12] | 9.1 (2.7) | 9.2 (2.6) | 8.2 (3.1) | 0.38 |
| Number of drugs | 5.1 (2.7) | 5.2 (2.7) | 4.2 (1.3) | 0.53^b^ |
| CIRS-G score [0-56] | 6.4 (2.1) | 6.2 (0.8) | 6.4 (2.2) | 0.86 |
| Deceased % (N) | 18.4 (18) | 18.9 (17) | 12.5 (1) | 1.00^a^ |
| Nursing-home admission % (N) | 19.4 (19) | 20.0 (18) | 12.5 (1) | 1.00^a^ |

**Supplementary table: Comparison of the characteristics of patients included in the multivariate analysis vs those excluded**

a : Fisher’s exact test ; b : Mann-Whitney test ; c : Missing values = 5/8

**Abbreviations**: ADL = Activities of Daily Living CIRS-G = Cumulative Illness Rating Scale for Geriatrics; IADL = Instrumental Activities of Daily Living; M = mean; MMSE = Mini Mental State Evaluation; N = number of subjects; SD = standard deviation; TUG = Timed Up and Go test; TUGc = Timed Up and Go Cognitive task; UPDRS = United Parkinson’s Disease Rating Scale
